# Subcellular Targeting Domains of Sphingomyelin Synthase 1 and 2

**DOI:** 10.1186/1743-7075-8-89

**Published:** 2011-12-14

**Authors:** Calvin Yeang, Tingbo Ding, William J Chirico, Xian-Cheng Jiang

**Affiliations:** 1 Department of Cell Biology, State University of New York Downstate Medical Center, 450 Clarkson Ave., Brooklyn, New York, 11203 USA; 2 Department of Biochemistry, School of Pharmacy, Fudan University, 826 Zhangheng Road, Shanghai, 5198000, China

## Abstract

Sphingomyelin synthase (SMS) sits at the crossroads of sphingomyelin (SM), ceramide, diacylglycerol (DAG) metabolism. It utilizes ceramide and phosphatidylcholine as substrates to produce SM and DAG, thereby regulating lipid messengers which play a role in cell survival and apoptosis. Furthermore, its product SM has been implicated in atherogenic processes such as retention of lipoproteins in the blood vessel intima. There are two mammalian sphingomyelin synthases: SMS1 and SMS2. SMS1 is found exclusively in the Golgi at steady state, whereas SMS2 exists in the Golgi and plasma membrane. Conventional motifs responsible for protein targeting to the plasma membrane or Golgi are either not present in, or unique to, SMS1 and SMS2. In this study, we examined how SMS1 and SMS2 achieve their respective subcellular localization patterns. Brefeldin A treatment prevented SMS1 and SMS2 from exiting the ER, demonstrating that they transit through the classical secretory pathway. We created truncations and chimeras of SMS1 and SMS2 to define their targeting signals. We found that SMS1 contains a C-terminal Golgi targeting signal and that SMS2 contains a C-terminal plasma membrane targeting signal.

## Introduction

Sphingomyelin synthase is the last enzyme required for de novo synthesis of sphingomyelin (SM). There are three isoforms of sphingomyelin synthase (SMS): SMS1, SMS2, and SMS related protein (SMSr). SMS1 and SMS2 are true SM synthases in that they both utilize phosphatidylcholine (PC) and ceramide to produce SM and diacylglycerol (DAG). Although SMSr is highly homologous to SMS1 and SMS2, it does not have SM synthase activity [1]. Instead, it regulates cellular ceramide levels through synthesis of ceramide phosphoethanolamine [2].

SMS1 and SMS2 are in a unique position to regulate cellular SM, ceramide, and DAG levels. SM, in addition to functioning as a structural component in biological membranes, preferentially interacts with cholesterol to form specialized membrane microdomains or "lipid rafts" [3]. Both ceramide and DAG have been implicated in numerous cell functions including growth, differentiation, signal transduction, proliferation, and apoptosis [4,5]. SMS1 and SMS2 differ, however, in their subcellular localization. At steady state, SMS1 resides in the Golgi, while SMS2 is located in the Golgi and plasma membrane. Flag-tagged SMS1 [6] and V5-tagged SMS1 [1] are located in the Golgi while flag-tagged SMS2 [6] and V5-tagged SMS2 [1] are found on the plasma membrane and in the Golgi. Furthermore, SMS1 knockdown in Hela cells attenuates SM synthase activity in the Golgi while SMS2 siRNA treatment in Hela cells reduces SM synthase activity in the plasma membrane [7]. These data support the validity of using epitope tagged SMS to study their localization patterns.

We examined how SMS1 and SMS2 are differentially targeted using GFP fusion proteins. Analysis of the primary sequence of SMS1 and SMS2 (Additional File 1, Figure S1), revealed that targeting of these proteins by conventional sorting signals is unlikely. There is no well characterized targeting signal which is unique to SMS1 or SMS2 explaining the difference in their localization patterns. Although transmembrane domains can dictate where a protein is targeted [8], SMS1, SMS2, and SMSr are predicted to have very similar transmembrane domains (Additional File 1, Figure S1), making that mechanism less favorable. Our approach was to manipulate regions of amino acid sequence differences between SMS1 and SMS2 and observe how those changes affect their localization. Our findings show that the C-terminus of SMS1 contains a Golgi targeting signal and that of SMS2 contains a signal for plasma membrane targeting.

## Materials and Methods

### Plasmid preparation

SMS1 (NM_147156), SMS2 (NM_028943), SMSr, (NM_026283) were purchased from Open Biosystem. The cDNA of SMS1 and SMS2 were cloned into pEGFP-C3 using PCR to amplify the coding region and simultaneously to introduce a 5' EcoR1 restriction site and 3' BamH1 restriction site (Table 1). Due to the presence of a BamH1 restriction site in the SMSr cDNA, SMSr was cloned using PCR primers coding for a 5' Hind III restriction site and a 3' EcoR1 restriction site. Truncation mutants were similarly constructed using PCR and all inserts had 5' EcoR1 restriction site and 3' BamH1 restriction site unless otherwise noted. The primers used to generate these mutants are listed in the Table 1. The CD4 containing plasmid was a generous gift from Dr. Sreenivasan Ponnambalam from University of Leeds.

**Table 1:** Primers used for PCR cloning

Construct name	Forward primer	Reverse Primer
GFP SMS1	GGA ATT CCG AAG GAA GTG GTT TAT TGG TC	CGG GAT CC T CAC CGG GAA TAC TTT CTG
GFP SMS2	GGA ATT CCG GAT ATC ATA GAG ACA GCA AAA C	CGG GAT CCT CAG GTA GAC TTC TCA TTA TCC TC
GFP SMSr	CCC AAG CTT CCC GCT GGT AGC CGA	CGG AAT TCC GTC CAA TTA GTC TTT TCA TTA TTG
GFP SMS1 N120	GGA ATT CCG CCA GAA CTG GAG CGC	CGG GAT CC T CAC CGG GAA TAC TTT CTG
GFP SMS1 N130	CGG AAT TCC GGA GTG GGG CAA GAC TTT TC	CGG GAT CC T CAC CGG GAA TAC TTT CTG
GFP SMS2 N20	CGG GAT CC T CAG GTA GAC TTC TCA TTA TCC TC	CGG GAT CCT CAG GTA GAC TTC TCA TTA TCC TC
GFP SMS2 N40	GGA ATT CCG AAA CCC AAG ACC TTA TCC	CGG GAT CCT CAG GTA GAC TTC TCA TTA TCC TC
GFP SMS2 N60	GGA ATT CCG CAG ATT TCC ATG CCC AAC	CGG GAT CCT CAG GTA GAC TTC TCA TTA TCC TC
GFP SMS2 N69	GGA ATT CCG AAC AAG TTT CCC CTG G	CGG GAT CCT CAG GTA GAC TTC TCA TTA TCC TC
GFP SMS1ΔC4	GGA ATT CCG AAG GAA GTG GTT TAT TGG TC	CGC GGA TCC CAG CCG GCT GTA TTT AA
GFP SMS1ΔC17	GGA ATT CCG AAG GAA GTG GTT TAT TGG TC	CGC GGA TCC TGG CCA GGG GAA AGG
GFP SMS1ΔC27	GGA ATT CCG AAG GAA GTG GTT TAT TGG TC	CGC GGA TCC AGG TAC AAT TCC TTG GAC
GFP SMS2ΔC32	GGA ATT CCG GAT ATC ATA GAG ACA GCA AAA C	CGG GAT CCA AAG CAG CAA GGA ATT GAG
GFP SMS2ΔC67	GGA ATT CCG GAT ATC ATA GAG ACA GCA AAA C	CGG GAT CCT CAC CGG GAA TAC TTT CTG C
SMS1 GFP	GGA ATT CCG AAG GAA GTG GTT TAT TGG TC	CGG GAT CCT GTG TCA TTC ACC AGC
SMS2 GFP	GGA ATT CCG GAT ATC ATA GAG ACA GCA AAA C	CGG GAT CCG GTA GAC TTC TCA TTA TCC TC
CD4 GFP	GGA ATT CTT GCC ACC ATG AAC CGG GGA GTC C	CGG GAT CCA ATG GGG CTA CAT GTC TT

nS1/S2 and nS1/S2 plasmid preparation: A Sal1 restriction site was introduced to SMS1 at amino codon 89 and, correspondingly, codon 31 on SMS2 (Additional File 1, Figure S1) by site directed mutagenesis using a Kit from Strategene. This resulted in a silent mutation of GTA to GTC in both SMS1 and SMS2. By a similar method, a Pst1 restriction site was introduced to within codons 131-132 on SMS1 and codons 72-73 on SMS2. This resulted in a mutation of CTT CAG (SQ) to CTG CAG (LQ) and a mutation of AAC AAG (KF) to CTG CAG (LQ). Both vectors containing GFP-SMS1 and GFP-SMS2 were digested with both Sal1 and Pst1. The region flanked by Sal1 and Pst1 was excised from SMS1 and ligated into the vector containing GFP-SMS2. The resulting chimera (nS1/S2) was cloned into pEGFP-C3. A chimera of SMS1 with a swapped segment of SMS2 N-terminus (nS2/S1) was created in the same fashion.

S1/cS2 and S2/cS1 plasmid preparation: A chimera consisting of SMS1 from its N-terminus to amino acid 331 and SMS2 from amino acid 276 to its C-terminus (S1/cS2) was cloned into pEGFP-C3. This was done by site directed mutagenesis (Strategene), introducing an AatII site into SMS1 and SMS2. Codons 331 and 332 GTG GTG (VV) was mutated to GAC GTG (VI) in SMS1. Similarly, a chimera of SMS2 from its N-terminus to amino acid 275 and SMS1 from amino acid 332 to its C-terminus (S2/cS1) was created. Silent mutations of GTC ATC (VI) to GAC GTG (VI) were introduced to codons 275 and 276 of SMS2. To construct the chimeras, each mutant plasmid was digested with EcoR1, AatII, and BamH1. The 5' EcoR1 and AatII digestion product of SMS1 was then ligated with the 3' AatII and BamH1 digestion product of SMS2 and inserted into pEGFP-C3, creating S1/cS2. S2/cS1 was similarly constructed.

### Cell Culture and Transfections

Monolayer HeLa cells (from American Type Culture Collection) were grown in DMEM supplement with 10% FBS, penicillin/streptomycin, and 2 mM glutamine. Cells were grown to 50-60% confluency before transfection by lipofectamine 2000 (Invitrogen). For fluorescent imaging of GFP-tagged proteins, cells were seeded into 8 well chamber slides (BD Falcon) and transfected with 200 ng plasmid DNA in OptiMem (Invitrogen) for 24 hours. For Western Blot analysis of GFP-tagged proteins, cells were seeded into a 12 well plate (Corning) and transfected with 1000 ng plasmid DNA in OptiMem for 24 hours.

### Brefeldin A treatment

Cells were transfected with GFP-SMS1 or GFP-SMS2 for 16 hours and then treated with brefeldin A (5 μg/ml) for 8 hrs. The cells were washed 3 times in PBS and then fixed in 4% formaldehyde in PBS. In parallel, cells expressing GFP-SMS1 or GFP-SMS2 and treated with brefeldin A (5 μg/ml) for 8 hours were washed 6 times to remove brefeldin A, incubated for 3 hours at 37 C and 5% CO2, and then fixed.

### Confocal microscopy and immunocytochemistry

HeLa cells expressing GFP-tagged proteins were fixed in 4% formaldehyde PBS and then viewed directly or subjected to immunocytochemistry. For immunocytochemistry, cells were permeabilized with 0.1% Triton X-100 PBS for 10 minutes and then blocked in 3% BSA PBS for 1 hour. Primary antibodies used include: rabbit anti-calnexin (Abcam) 1:250, rabbit anti-TGN46 (Sigma) 1:250, and rabbit anti-pan-Cadherin (Abcam) 1:250. Cells were washed 3 times in PBS and incubated with anti-rabbit IgG conjugated to Alexa Fluor 540 for 1 hour. After three washes, TO-PRO 3 (Invitrogen), diluted 1:1000 in PBS, was added to cells to stain the nuclei. Slides were mounted in VectaShield (Vector Labs) and analyzed on a confocal microscope (Bio-Rad Radiance 2000) using 488 nm, 543 nm, and 638 nm excitation and a 40 X objective lens. Wheat germ agglutinin (WGA)- Alexa Fluor 647 (1:1000) was added to live cells prior to fixation to minimize intracellular staining.

## Results

### Localization of GFP-SMS1 and GFP-SMS2

**Figure 1:**
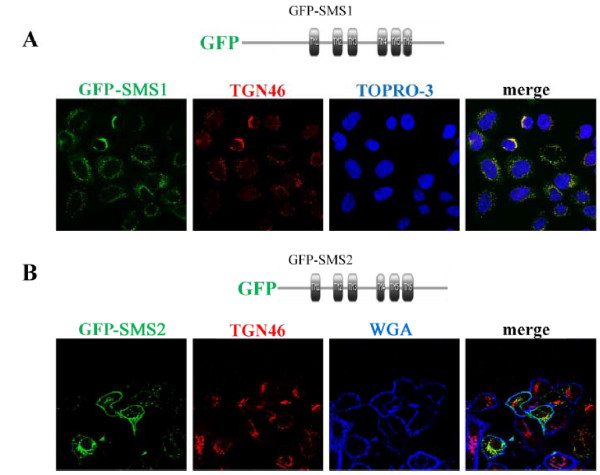
**Localization of GFP-SMS1 and 2 fusion proteins**. Confocal microscopy showing co-localization of TGN46 (Golgi marker) and Wheat Germ Agglutinin (WGA, plasma membrane marker) with GFP-SMS1 (A) and GFP-SMS2 (B). Schematics depict predicted transmembrane (TM) domains in SMS1 and SMS2. TOPRO is a marker for nucleus.

GFP expressed in mammalian cells is primarily cytosolic and widely used to study targeting signals. We prepared GFP fusion proteins of SMS1, SMS2, and SMSr to identify the domain(s) that are responsible for their sorting. GFP-SMS1 was co-localized with trans-Golgi network peptide 46 (TGN46) to the Golgi apparatus (Figure 1A) while GFP-SMS2 was localized to the Golgi apparatus and plasma membrane (Figure 1B). GFP-SMSr had a unique subcellular localization pattern, co-localizing with calnexin in the endoplasmic reticulum (ER) (Additional File 1, Figure S2). All fusion proteins were expressed at the expected molecular size, as determined by Western Blot (Additional File 1, Figure S3).

### Brefeldin A inhibits the sorting of SMS1 and SMS2

**Figure 2:**
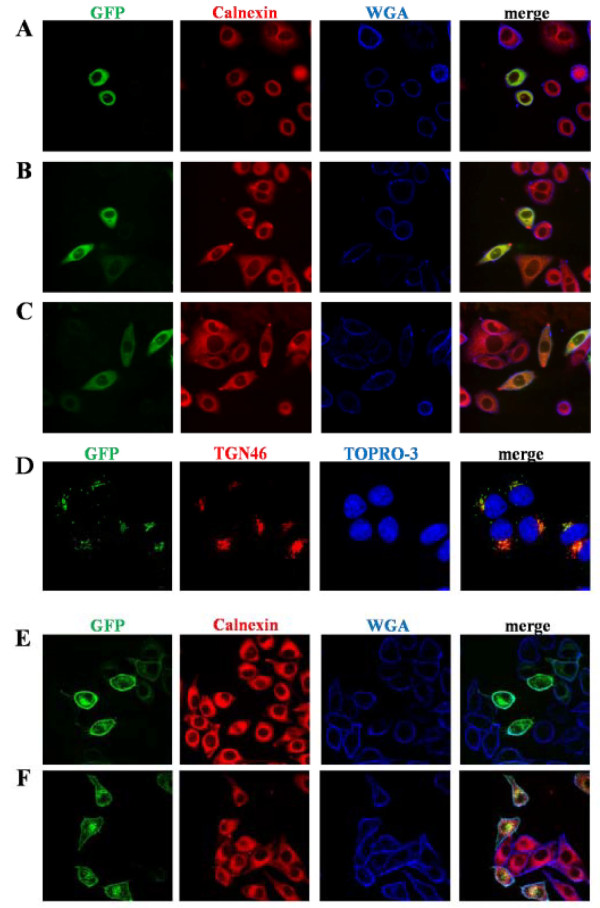
**BFA blocks sorting of SMS1 and 2**. HeLa cells expressing GFP-SMS1 (A and D), GFP-SMS2 (B and E), CD4-GFP (C and F) were treated with BFA (5 μg/ml) for 8 hours (A-C). The cells were washed 6 times with growth media to remove BFA and allowed to recover for 3 h (D-F). Images were obtained using confocal microscopy. Calnexin is an ER marker, TGN46 is a Golgi marker, and Wheat Germ Agglutinin (WGA) is a plasma membrane marker.

The fungal metabolite brefeldin A (BFA) inhibits anterograde vesicular transport from the ER to Golgi [9] and thereby leads to accumulation of proteins which transit through the classical secretory pathway in the ER. Brefeldin A reversibly blocks ER to Golgi transfer through inhibiting ARF, a small GTPase responsible for vesicle formation [10]. We treated HeLa cells expressing GFP-SMS1 or GFP-SMS2 with BFA to determine if they are sorted via the classical secretory pathway. CD4-GFP, an integral membrane protein which is sorted to the plasma membrane via the classical secretory pathway [11], was used as a control. In BFA-treated cells, GFP-SMS1 (Figure 2A), GFP-SMS2 (Figure 2B), and CD4-GFP (Figure 2C) localized to the ER. In contrast, GFP localization was unaffected by BFA (not shown). BFA can be removed from cells by washing, thereby restoring transport through the pathway. After removing BFA from cells expressing GFP-SMS1, GFP-SMS2 or CD4-GFP, the wild-type localization pattern of each protein was rescued (Figure 2D-F). Together, these results suggest that SMS1 and SMS2 are sorted through the classical secretory pathway.

### Probing SMS1's and SMS2's N-terminus for subcellular targeting signals

**Figure 3:**
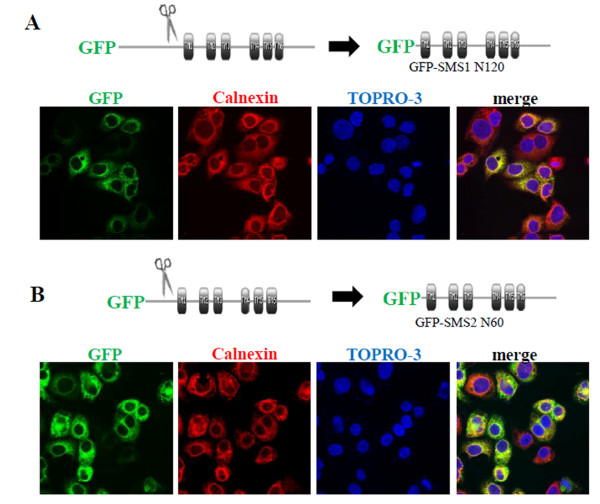
**Localization of N-terminal truncation mutants of SMS1 and SMS2**. HeLa cells expressing GFP-SMS1 N130 (A) and GFP-SMS2 N60 (B). The organelle markers used were calnexin (ER) and TOPRO 3 (nucleus). The images were obtained using confocal microscopy.

We examined the N-terminus of SMS1 and SMS2 for sorting signals because this region contained the most sequence differences and might be responsible for the different localization patterns. Truncation of 120 amino acids from the SMS1 N-terminus (GFP-SMS1 N120) and truncation of 40 amino acids from the SMS2 N-terminus (GFP-SMS2 N40) did not affect the localization pattern of these proteins (Additional File 1, Table S1). However, truncation of 130 amino acids from SMS1 (GFP-SMS1 N130) and 60 amino acids from SMS2 (GFP-SMS2 N60) resulted in an ER and preinuclear localization pattern (Figure 3 and Additional File 1, Table S1).

**Figure 4:**
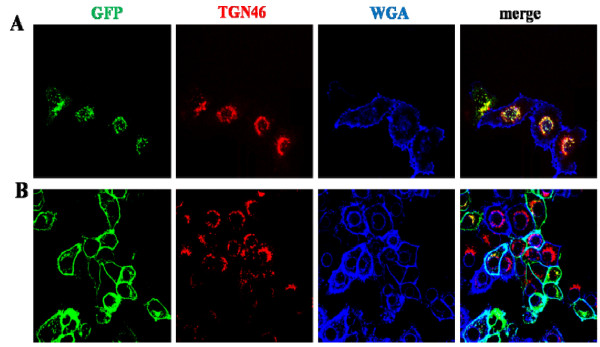
**Localization of SMS1 and SMS2 N-terminal chimeras**. HeLa cells transfected with GFP-nS2/S1 (A) or GFP-nS1/S2 (B). The organelle markers were TGN46 (*trans*-Golgi) and WGA (plasma membrane). Images were obtained using confocal microscopy.

It was possible that these truncations disrupted or removed a signal that was important for the normal targeting of SMS1 and SMS2. However, we also considered the possibility that these truncations can lead to misfolding of the protein and thereby induce chaperone mediated retention in the ER. Unlike truncations, domain exchange better preserves the overall architecture of the protein. Therefore, to further study the N-terminus of SMS1 and SMS2, we created chimeras by swapping regions of the N-terminus between SMS1 and SMS2. The region in SMS1 extends from amino acid 121 to 131 (where sequence homology with SMS2 resumes). Similarly, the region in SMS2 extends from amino acid 61 to 77. A chimera of SMS1 with a portion of SMS2's N-terminus (nS2/S1) and one of SMS2 containing a portion of SMS1's N-terminus (nS1/S2) were created. nS2/S1 localized to the Golgi only (Figure 4A), as does full length SMS1 (Figure 1A), while nS1/S2 localized to the Golgi and plasma membrane (Figure 4B), as does full length SMS2 (Figure 1B). Therefore, the N-terminus of SMS1 does not contain a Golgi targeting signal and the N-terminus of SMS2 does not contain a plasma membrane targeting signal.

### The C-terminus of SMS1 contains a Golgi retention signal and C-terminus of SMS2 contains a plasma membrane targeting signal

**Figure 5:**
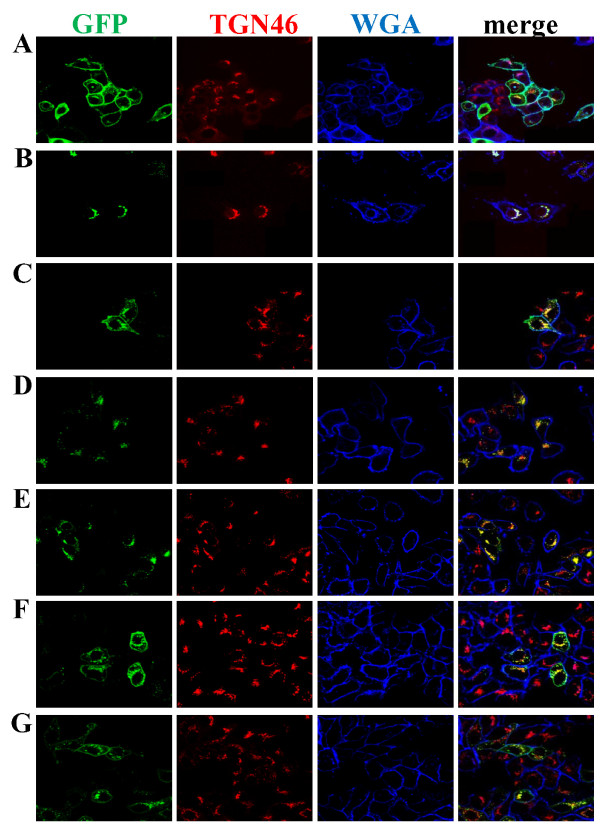
**Localization of SMS1 and SMS2 C-terminal chimeras and mutants**. Confocal images of HeLa cells transfected with A) GFP-S1/cS2, B) GFP-S2/cS1, C) GFP-S2ΔC32, D) GFP-S2ΔC67, E) GFP-S1ΔC4, F) GFP-S1ΔC17, and G) GFP-S1ΔC27. Organelle markers used were TGN46 (Golgi) and WGA (plasma membrane).

We also examined the C-terminus of SMS1 and SMS2 for sorting signals because they contain several amino acid sequence differences that might be responsible for differences in their subcellular localization. Using the same approach described in the previous section, we created chimeras by exchanging the C-terminus of SMS1 and SMS2 yielding S1/cS2 and S2/cS1. S1/cS2 is a GFP fusion composed of residues 1-331 of SMS1 and residues 276-356 of SMS2. S2/cS1 is a GFP fusion composed of residues 1-275 of SMS2 and residues 332-414 of SMS1. S1/cS2 localized to the plasma membrane and Golgi (Figure 5A), which matches the wild type SMS2 pattern. S2/cS1 localized to the Golgi only (Figure 5B), mimicking wild type SMS1. These results suggest that SMS1 possesses a Golgi retention or retrieval signal in the C-terminus and that SMS2 has a plasma membrane targeting signal in its C-terminus.

To better define the signal, we created C-terminal truncation mutants of SMS1 and SMS2. SMS2 localized to both the plasma membrane and Golgi when its most distal 32 amino acids were removed (S2ΔC32, Figure 5C). In contrast, SMS2 localized only to the Golgi when 67 amino acids from its C-terminus were removed (S2ΔC67, Figure 5D). Deleting the 4 most distal amino acids from SMS1 did not change its native localization pattern (S1ΔC4, Figure 5E). However, deleting 17 (S1ΔC17, Figure 5F) or 27 amino acids (S1ΔC27, Figure 5G) from the C-terminus of SMS1 resulted in a plasma membrane and Golgi targeted protein. These data suggest that SMS1 has a Golgi retention or retrieval sequence between amino acid residues 332 and 410.

## Discussion

In this study, we found for the first time that SMS1 contains a C-terminal Golgi targeting signal, while SMS2 contains a C-terminal plasma membrane targeting signal. The difference in cellular localization may reflect the distinct functions of SMS1 and SMS2. Given their role as a potential regulator of diseases, these findings, coupled with our homology modeling of SMS1 and SMS2 [6], will be useful for developing SMS1- and SMS2-specific inhibitors.

SMS1 and SMS2 transit through the classical secretory pathway and their respective C-terminal tails determine the fate of localization along the secretory pathway. Recently, Vacaru et al. [2] demonstrated that truncation of the SMSr N-terminus results in Golgi localization of the protein, suggesting that SMSr also enters the classical secretory pathway.

Proteins destined for the classical secretory pathway generally contain an N-terminal ER signal sequence which facilitates the proteins co-translational insertion into the ER [12]. Although a signal sequence is absent in SMS1 and SMS2, not all proteins which enter the ER have a signal sequence. Examples are multipass transmembrane proteins such as opsin, rhodopsin and other G-protein coupled receptors (GPCRs)[13]. Insertion of opsin into the ER membrane is guided solely by multiple hydrophobic internal start- and stop-transfer sequences within the protein and dependent on SRP [14]. Following incorporation into the ER, these proteins can be further sorted via vesicular transport to organelles including the Golgi, plasma membrane (as described below), lysosome, and endosome. It is likely that each of the predicted hydrophobic transmembrane domains within SMS1 and SMS2 act as internal start- and stop-transfer sequences.

Once inside the classical secretory pathway, the C-terminus of SMS1 and SMS2 direct the protein to its steady state location. A chimera of SMS2 containing the SMS1 C-terminus is sorted to the Golgi, while a chimera of SMS1 containing the SMS2 C-terminus is released to the plasma membrane. We demonstrated that the SMS1 C-terminus contains a Golgi targeting signal which can be disrupted by truncating its last 17 amino acids. In the absence of the Golgi targeting signal, it's likely that SMS1 proceeds through the secretory pathway to reach the plasma membrane. Bos et al. [15] have shown that TGN 38, a Golgi targeted protein, is localized to the plasma membrane if its Golgi targeting signal is disrupted. Similarly, SMS1 contains a retention or retrieval signal which prevents it from being localized to the plasma membrane. SMS2 contains a C-terminal plasma membrane targeting signal which can be disrupted by truncating the last 67, but not 32 amino acids. In the absence of this signal, SMS2 is found in the Golgi, which suggests the existence of a Golgi targeting sequence which is masked by the presence of the last 67 amino acids.

Tani and Kuge reported that SMS2 is specifically palmitoylated on four serine residues at the C-terminus, and this palmitoylation is involved in SMS2 plasma membrane localization [16]. We observed that S2ΔC32 (which still contains palmitoylation sites C331, C332) does not affect plasma membrane localization while S2ΔC67 (which has no palmitoylation sites) fails to localize to the plasma membrane (Figure 5C and 5D). These findings are consistent with the role of palmitoylation in targeting SMS2 to the plasma membrane. However, the C331, C332, C343, and C348 quadruple mutant of SMS2 was found to have less plasma membrane localization compared to WT SMS2 [16] while S2ΔC67 is not found on the plasma membrane (Figure 5D), suggesting that the SMS2 C-terminus contains multiple signals important for plasma membrane targeting. However, we could not rule out the possibility that the half life of SMS2ΔC67 on the plasma membrane is greatly decreased or removal of the SMS2 C-terminal segment produces a mutant which is more susceptible to degradation at the plasma membrane and therefore is predominantly located in the Golgi at steady state. We believe that this is an interesting issue which deserves further study.

In this study, which focuses on the subcellular localization of SMS1 and SMS2, we utilized GFP-tagged SMS, which can be directly visualized in the cell using confocal microscopy. Although it is possible that GFP, as an N-terminal fusion protein, may influence sorting of these proteins, it is unlikely based on existing evidence. SMS1 containing a C-terminal Flag tag [6] or C-terminal V5 tag [1] localize to the Golgi, as does GFP-SMS1. Similarly, SMS2 containing a C-terminal Flag tag [6] or C-terminal V5 tag [1] localize to the Golgi and plasma membrane, as does GFP-SMS2. GFP is a widely used tool to study protein sorting, and it is justifiably used to exam the subcellular targeting of SMS1 and SMS2.

Our results shed new light on the signals that guide SMS1 and SMS2 to their steady state sub-cellular compartments. Further study manipulating the localization of these enzymes may reveal the importance of subcellular compartment-specific SM synthesis.

## Competing interests

The authors declare that they have no competing interests.

## Authors' contributions

CY participated in the design of the study, performed the experiments, and drafted the manuscript. TD carried out SMS measurement and modified the manuscript. WJC participated in the design of the study and modified the manuscript. XCJ conceived of the study, participated in its design and coordination, and drafted the manuscript. All authors read and approved the final manuscript.

## Supplementary Material

Additional file 1 **Supplemental Data**. Supplemental Data and Legends. Click here for file
