# Supplementary material for: Subcellular Targeting Domains of Sphingomyelin Synthase 1 and 2
Source: Nutr Metab (Lond). 2011 Dec 14;8:89. doi: 10.1186/1743-7075-8-89 (PMC3264500; doi:10.1186/1743-7075-8-89)

Supplement

Supplemental Table I. SMS1 and SMS2 truncational mutants and their respective localization pattern.

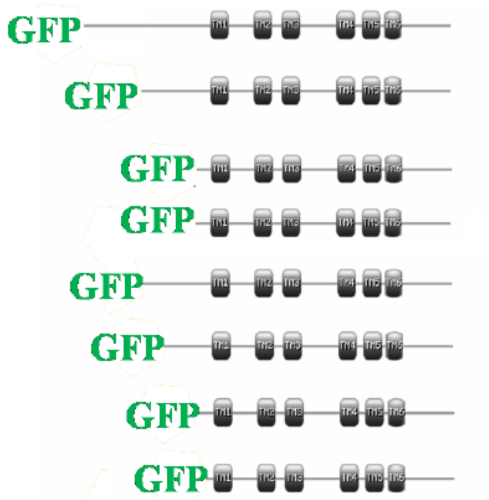

	Plasma Membrane	Golgi	ER / perinuclear
SMS1		X	
SMS2	X	X	
SMS1 N120		X	
SMS1 N130			X
SMS2 N20	X	X	
SMS2 N40	X	X	
SMS2 N60			X
SMS2 N69			X

Supplemental Figure I. Sequence alginment of SMS1, SMS2, and SMSr. Bold lines indicate predicted transmembrane domains. Sequences were aligned using CLUSTALW and transmembrane domains predicted using TMHMM.

Supplemental Figure II. Localization of GFP-SMSr fusion proteins. Confocal microscopy showing co-localization of calnexin (ER marker), TGN46 (Golgi marker), and Wheat Germ Agglutinin (WGA, plasma membrane marker) with GFP-SMSr. Schematics depict predicted transmembrane (TM) domains in SMSr.

Supplemenal Figure III. Western blot of all fusion proteins. All fusion protein were expressed at the expected molecular size.

Supplemental Figure I. Yeang et al.

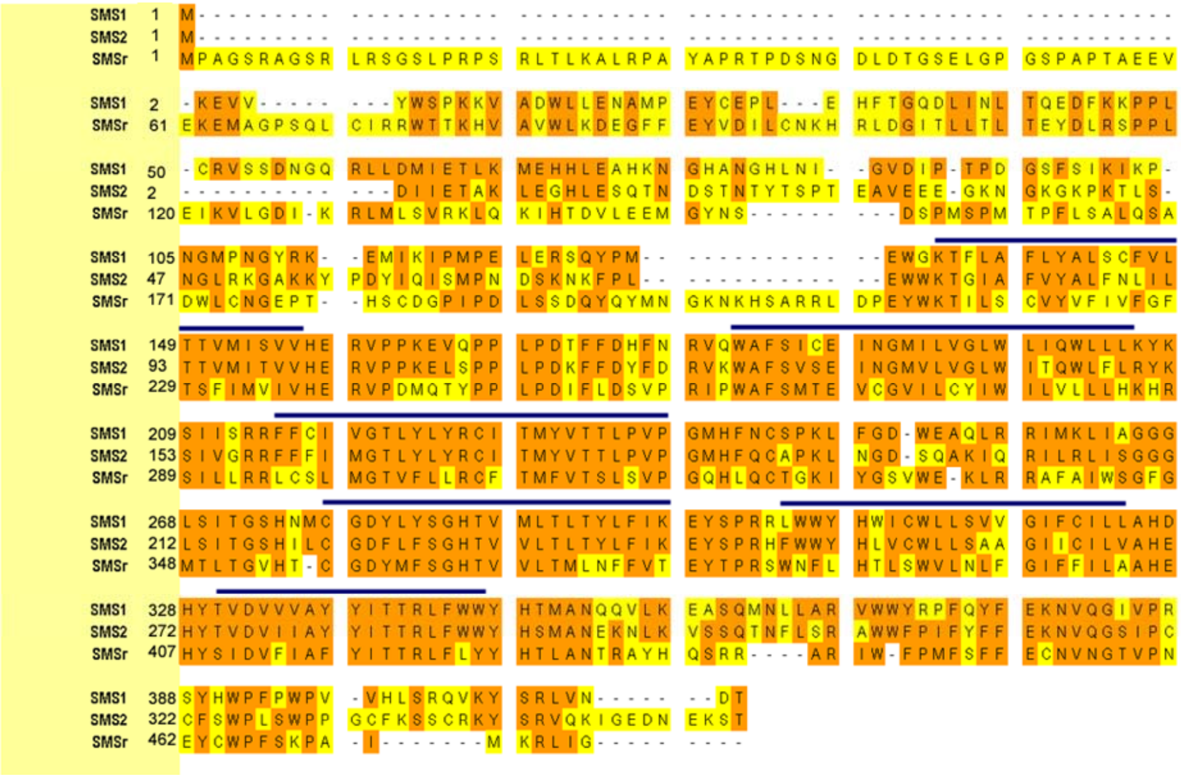

Supplemental Figure II. Yeang et al.

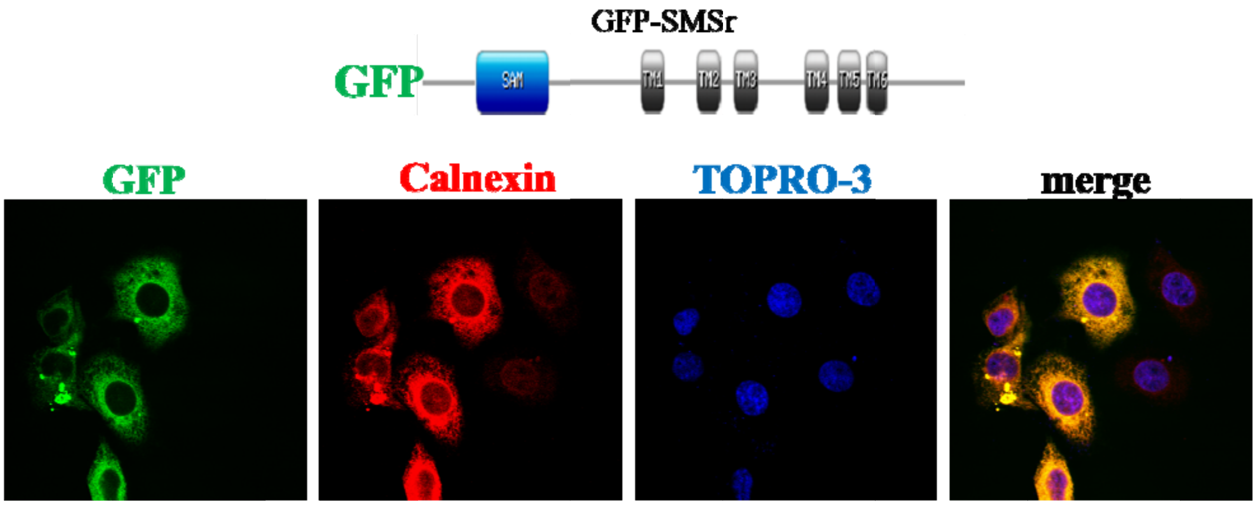

Supplemental Figure III. Yeang et al.

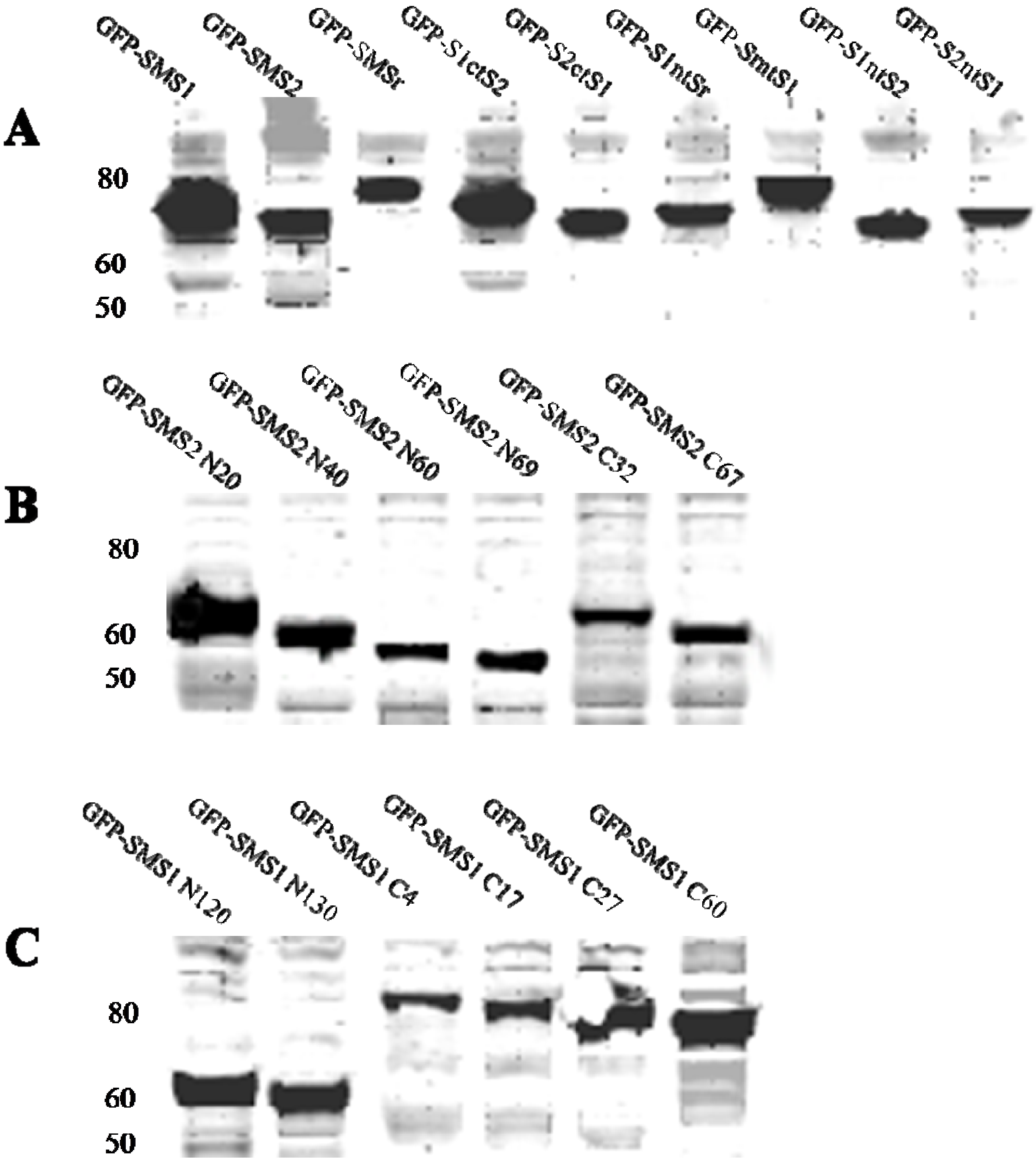

Supplement: Additional file 1 — Supplemental Data. Supplemental Data and Legends. [file 1743-7075-8-89-S1.PDF]
